# Youth and parent perceptions on participating in specialized multidisciplinary pain rehabilitation options: A qualitative timeline effect analysis

**DOI:** 10.1080/24740527.2020.1858709

**Published:** 2021-02-03

**Authors:** Karen Hurtubise, Astrid Brousselle, Melanie Noel, Abbie Jordan, Jo White, Nivez Rasic, Chantal Camden

**Affiliations:** a Faculté de Médecine et Sciences de la Santé, Université de Sherbrooke, Sherbrooke, Québec, Canada; b School of Public Administration, University of Victoria, Victoria, British Columbia, Canada; c Department of Psychology, University of Calgary, Calgary, Alberta, Canada; d Hotchkiss Brain Institute, Health Research Innovation Centre, Calgary, Alberta, Canada; e Department of Psychology and Centre for Pain Research, University of Bath, Bath, UK; f Department of Health and Social Sciences, University of the West of England–Bristol, Bristol, UK; g Department of Anesthesia & Pain Medicine, Foothills Hospital, Calgary, Alberta, Canada; h CanChild Centre for Childhood Disability Research, McMaster University, Hamilton, Ontario, Canada

**Keywords:** pediatric pain-related disability, specialized pain rehabilitation, treatment experience, timelines, qualitative method

## Abstract

**Background**: Little is known about how the specialized treatment journey is perceived by youth with pain-related disability and their parents.

**Aims**: Describe and compare the treatment effects and outcomes as perceived by youth and their parents enrolled in intensive interdisciplinary pain treatment (IIPT) or multimodal treatment (MMT).

**Methods**: Eleven IIPT youth and five parents and three MMT youth and five parents were recruited. All were asked to complete a treatment journey timeline, followed by separately conducted semistructured interviews. Transcribed interviews were analyzed using reflective thematic analysis.

**Results**: The main themes spanned the treatment trajectory. All participants described similar initial struggles (Theme 1). Positive and negative treatment effects associated with acquisitions and disruptions (Theme 2), and outcomes post-discharge related to supports and realities (Theme 3) emerged. Knowledge, skills, and support acquisition during treatment and feeling empowered and confident to self-manage postdischarge were identified as IIPT benefits. However, the change effort and life disruptions required and the difficulty transitioning to real life postprogram were acknowledged as detrimental IIPT impacts. Continuing with life as usual and maintaining supports in daily contexts (e.g., school personnel, friends) were reported MMT benefits. However, the challenges of managing pain, treatment adherence within the competing demands of daily realities, and the lack of support to integrate strategies were emphasized as detrimental MMT impacts.

**Conclusions**: Detailed impacts of two specialized multidisciplinary pain rehabilitation interventions on the lives of youth with pain-related disability and their parents are described. The treatments benefits and previously unexplored detrimental effects are unveiled.

## RÉSUMÉ

**Contexte:** On en sait peu sur les parcours de traitement spécialisés tels que perçus par les jeunes ayant une incapacité liée à la douleur et par leurs parents.

**Objectifs:** Décrire et comparer les effets et les résultats du traitement tels qu’ils sont perçus par les jeunes inscrits à un traitement interdisciplinaire intensif de la douleur ou à un traitement multimodal, ainsi que par leurs parents.

**Méthodes**: Onze jeunes inscrits à un traitement interdisciplinaire intensif de la douleur et cinq parents, ainsi que trois jeunes inscrits à un traitement multimodal et cinq parents, ont été recrutés. Tous ont été invités à décrire le calendrier de leur parcours de traitement, puis à participer à des entrevues semi-structurées menées séparément. Les entrevues transcrites ont été analysées à l’aide d’une analyse thématique réflexive.

**Résultats**: Les principaux thèmes couvraient la trajectoire du traitement. Tous les participants ont décrit des difficultés initiales similaires (Thème 1). L’association des effets positifs et négatifs du traitement avec les acquis et les perturbations (Thème 2), et les issues liées au soutien et aux réalités suite à leur congé (Thème 3) ont émergé. L’acquisition de connaissances et d’habiletés, l’accès à du soutien et le sentiment d’autonomisation et de confiance en sa capacité d’autoprise en charge après le congé ont été répertoriés comme des avantages du traitement interdisciplinaire intensif de la douleur. Toutefois, les efforts de changement requis et les perturbations de la vie nécessaires, ainsi que les difficultés de la vie réelle après le programme, ont été considérés comme des répercussions négatives du traitement interdisciplinaire intensif de la douleur. La possibilité de poursuivre la vie comme d’habitude et de conserver le soutien dans des contextes de la vie quotidienne (ex. : personnel scolaire, amis) ont été rapportés comme des avantages du traitement multimodal. Toutefois, les défis liés à la prise en charge de la douleur, l’adhésion au traitement dans le cadre des exigences concurrentes des réalités quotidiennes, ainsi que le manque de soutien pour intégrer les stratégies, ont été mis en relief comme des effets néfastes du traitement multimodal.

**Conclusions**: Les effets détaillés de deux interventions multidisciplinaires spécialisées en matière de réadaptation pour la douleur dans la vie de jeunes atteints d’une incapacité liée à la douleur et de leurs parents ont été décrits. Les avantages des traitements et leurs effets néfastes qui n’avaient pas encore été étudiés ont été dévoilés.

## Introduction

Pediatric chronic pain is a complex medical issue. For a clinically important subset of youth, it results in severe dysfunction and worsening disability affecting their physical, emotional, and social well-being.^1–5^ These youth experience challenges related to social development, peer interactions, and family functioning.^6–9^ Parents of these youth report emotional distress, helplessness, and altered parenting experiences.^5,10–12^ Because of the complexity of the pediatric chronic pain experience, comprehensive treatments, grounded in a biopsychosocial model and involving the expertise of an array of health care disciplines, are required.^13^ Specialized multidisciplinary pain rehabilitation, including outpatient multimodal treatment (MMT), inpatient or day hospital intensive interdisciplinary pain treatment (IIPT), is supported as the treatment choice.^14,15^ MMT consists of an amalgamation of medical (e.g., medications), physical (e.g., physical therapy, occupational therapy), and psychological interventions (e.g., cognitive behavioral therapy).^15,16^ IIPT consists of a defined period (e.g., 4 weeks) of intensive daily physical, occupational, and psychological therapies, along with medical support focused on functional restoration and self-management.^13,17^

The literature surrounding the evaluation of these treatments is expanding. To date, findings have relied on quantitative focused effectiveness studies, using quasi-experimental, nonrandomized cohort designs without a control group. These studies entail administering a battery of self-report questionnaires to program participants at various time points, typically at baseline, discharge, and shortly thereafter (e.g., 3 months).^13^ Recently, the relevance of some of these outcome domains (e.g., pain intensity) has been called into question by stakeholders, and the lack of appropriate tools to measure some of these domains has been underscored.^18^ Despite promising results demonstrated by these quantitative studies, many questions remain unanswered. For example, little is known about why some youth beneﬁt more than others, why adherence to therapeutic recommendations can be problematic, and what aspects of treatment promote long-term benefits. More important, the negative effects, outcomes, and impacts of specialized multidisciplinary pain rehabilitation contexts have yet to be explored in the current literature, limiting the knowledge associated with iatrogenic effects.

To address these unanswered questions and to increase our understanding of specialized multidisciplinary pain rehabilitation, rigorous qualitative studies are required.^19,20^ Few studies have targeted the treatment experiences of youth with pain-related disability^7,19,20^ or those of their parents.^21^ No studies have yet, to our knowledge, applied a qualitative narrative temporal approach and explored the postdischarge outcomes and longer-term impacts of specialized multidisciplinary pain rehabilitation or compared the differences between the various treatment options. This study aimed to explore the treatment effects, outcomes, and impacts as perceived and experienced by youth with pain-related disability and their parents at least 1 year following participation in a specialized multidisciplinary pain rehabilitation treatment option (i.e., either IIPT or MMT) and compare them.

## Materials and Methods

This study was part of a larger participatory program evaluation whose purpose was to assist decision makers in determining the future of an IIPT at a pediatric facility in Western Canada. This effect analysis study aimed to highlight all of the treatment effects, whether positive or negative, and whether they were attributable to the program. Effect analysis is a type of evaluation design that aspires to uncover all of the effects associated with an intervention, including those less explored and/or perceived as negative.^22^

### Study Context

Three partner organizations are involved in the Complex Pain Program provision of care: (a) a tertiary care pediatric health and rehabilitation facility; (b) a province-wide publicly funded health care organization; and (c) a specialized school, located within the walls of the tertiary care facility, yet part of the regional board of education. The family-centered care philosophy unifies the organizations. The Complex Pain Program includes comprehensive multidisciplinary specialty clinics, associated outpatient MMT services (e.g., psychology, physiotherapy, medical interventions, psychoeducation), and a day hospital IIPT. Youth with pain-related disability assessed by the specialty clinics can avail of the MMT and/or the IIPT interventions. In addition to coordination and administrative staff, the program incorporates a comprehensive interdisciplinary team (i.e., physicians, nurses, psychologists, physiotherapists, and a family counselor) trained in pediatric pain. Furthermore, it shares a staff complement with the rehabilitation day hospital services (e.g., recreation, occupational therapist, and program coordinator) and facility-wide services (i.e., teachers, spiritual care, art and music therapy). The assistant manager of rehabilitation services oversees the allied health and nursing human resources.

Stakeholders within a program context can challenge the establishment of common evaluation goals.^23^ As part of the overall participatory study, a 13-member advisory committee composed of youth with chronic pain-related disability, their parents, and other important stakeholders (i.e., a physiotherapist, an occupational therapist, a psychologist, a clinic nurse specialist, a physician, two health care managers, and two teachers) was involved in key decisions throughout the study cycle. As part of these decisions, the advisory committee completed a consensus-building exercise where six outcome domains were prioritized for measurement in the effect analysis.^18^ These outcome domains included participation in meaningful activities, activities of daily living, mood and affect, roles and relationships, school engagement, and self-efficacy.

### Study Design

An interpretive descriptive methodology was adopted. This qualitative research design uses an analytical, inductive approach that aims to uncover new ways of understanding human health and aspects related to the experience of disease that impact the clinical context and the practice of applied health disciplines.^24^ This methodology was chosen to gain insight into the way youth with pain-related disability and their parents described the effects (short term), outcomes (medium term), and impacts (long term) of participating in one of two specialized multidisciplinary rehabilitation pain treatment options and whether differences between the treatments were acknowledged.

### Participants

A purposive sample of youth with chronic pain and their parents was recruited through the complex pain clinics. Purposeful sampling is widely used in qualitative research for the identification of information-rich cases and is an effective use of limited resources.^25^ More specifically, we aimed to ensure representation of males, participants who were or whose parents were separated or divorced, and those of lower socioeconomical status to represent voices of people with lived experience who have been underrepresented in previous IPPT and MMT studies.

Potential participants were excluded if they were in an acute diagnostic stage where all “organic” or disease-related causes for their pain had not been reasonably ruled out (e.g., cancer) or if they presented with a psychological condition for which admission to a specialized psychiatric program at our facility was recommended by the clinical team (i.e., physician, psychologist, social worker, clinical nurse specialist, physiotherapist). These conditions included active psychosis or suicidal ideation, severe depression, or an eating disorder. Youth were eligible to participate if they were 12 to 18 years of age (i.e., the age range of the IIPT program), could follow verbal instructions in English, had no underlying disease that could explain their pain, had reported pain for at least 3 months (in accordance with the definition of chronic pain endorsed by the International Association for the Study of Pain),^26^ and, when screened by the clinical team, met the established pain-related disability criteria (i.e., repeated school absenteeism, withdrawal from leisure and sporting activities, and/or difficulty with mobility, daily hygiene, or other activities of daily living). Youth were required to have participated in one of two specialized multidisciplinary treatment options at the facility; that is, either IIPT or MMT. Parents were eligible to participate if they were the youth’s legal guardian. No other formal parent screening occurred.

**Figure 1.**
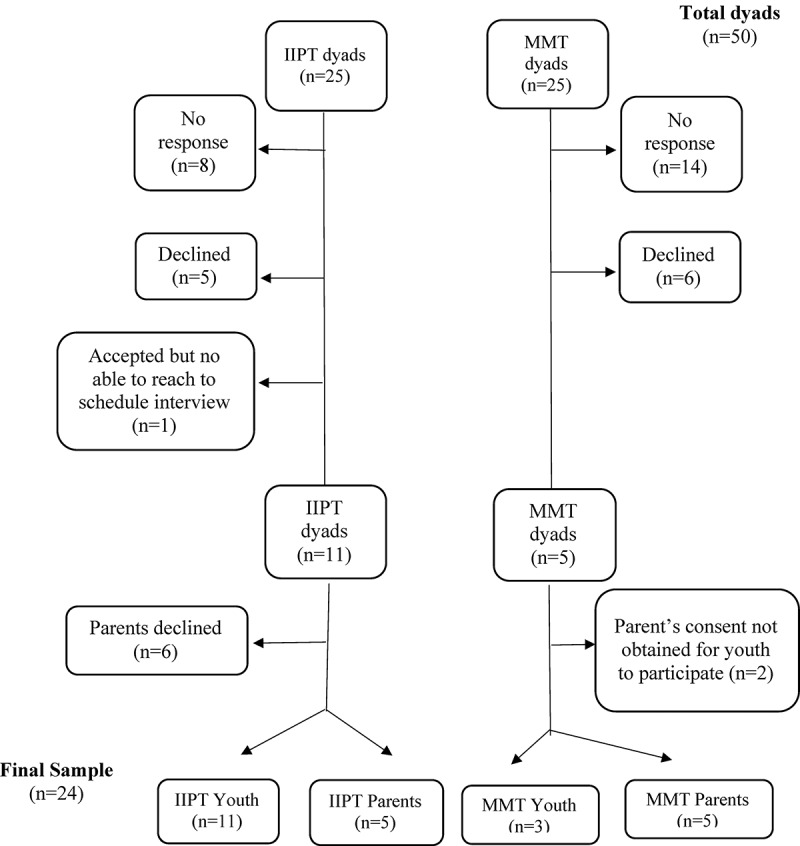
Participant recruitment. Total number of parent and youth dyads approached to participate, how the study sample was generated, and reasons for exclusion

All parent and youth dyads (25 parents and 25 youth who completed the IIPT and 25 parents and 25 youth involved in the MMT) who met the aforementioned criteria, agreed to participate in research at their initial clinic team visit, and completed the battery of self-assessment questionnaires at two time points following treatment, including at 12 months, were contacted (up to three times) by the research team using parent-provided contact information. As depicted in Figure 1, 68% of the potential sample did participate. Of those who did not, many did not respond (*n* = 22 dyads), others declined (*n* = 11 dyads), and another agreed to participate but could not be reached to schedule the interview (*n* = 1 dyad). Although limited time was a cited reason for declining to participate in both groups (IIPT *n* = 4; MMT *n* = 1), other reasons varied slightly between the treatment options. For IIPT, one youth was hospitalized for a mental health crisis (*n* = 1), and for MMT, frustrating experience with the program (*n* = 2), pursuit of other interventions (*n* = 1), parent hospitalization (*n* = 1), and participation in too many studies already (*n* = 1) were other cited reasons. Of the 19 remaining, 6 IIPT parents declined to participate themselves, yet their child agreed. Although most of these parents initially cited a lack of time to participant, many also acknowledged limited knowledge of their child’s pain status since the program, because it was now rarely a discussion topic. Conversely, in the MMT, two parents elected to participate instead of their child, suggesting that youth involvement in the study would remind the child of their pain or the negative experiences surrounding it or the child was seeking other treatment (e.g., surgery) to “resolve” their pain.

Twenty-four youth and parents were interested, deemed eligible, and completed, signed, and returned the online consent forms to the research team; 14 were youth (11 from IIPT and three from MMT), and 10 were parents (five from IIPT and five from MMT). Eight parent–youth dyads were present across our sample: five dyads representing the IIPT and three in the MMT. Youths’ (*M*_age_ = 17 years) predominant complaints were musculoskeletal and neuropathic pain (79%), yet some also presented with generalized pain (21%) and headache (14%). For most youth, symptoms had been present for over 12 months (93%). Table 1 provides additional socioeconomic (e.g., household income, marital status) and medical characteristics. When contextualizing our study, the sample characteristics present many similarities (e.g., sex, socioeconomic status, and marital status) to other qualitative studies in the field.^16,26–31^

**Table 1.** Participants’ demographic and pain characteristics

	IIPT	MMT
Youth Participants	N = 11	N = 3
Youth’s Median Age*, years (Range)	17 (12–18 years)	17 (12–18 years)
Youth’s Sex Female Male	10 1	3 0
Youth’s School Status Full Time Part-time On-line	9 1 1	1 1 1
Pain Location Musculoskeletal Neuropathic (e.g., CRPS) Headache Generalized	6 2 1 2	1 0 1 1
Pain Chronicity 6-12 months >12-months	1 10	0 3
Time since participation in program, months (mean)	24	19
Parent Martial Status Married Separated/Divorced	9 2	2 1
Parent Participants	N = 5	N = 5
Relationship to youth Biological mother Biological father Caregiver	4 1 0	4 0 1
Household income $30,000–$59,999 $60,000–$89,999 >$90,000 Do not want to answer	0 0 3 2	1 0 3 1
Marital Status Married Divorce/Separated	5 0	3 2

Legend: * related to participants age at the commencement of the program; CRPS= Complex Regional Pain Syndrome.

Although small, our sample size is also consistent with previous studies using inductive reflexive thematic analysis,^8,32,33^ those using qualitative methodology in the field of pediatric chronic pain,^8–10,21,34–37^ and those using timelines as the data collection approach.^38–42^ Our participants represented the diversity we aimed to achieve by our targeted sampling strategy. Further examination of our participant characteristics also highlighted participants in alternate custody arrangement (e.g., living with caregivers other than biological parents) and those identifying as First Nations/Indigenous Canadian.

Finally, instead of focusing on achieving data saturation,^27,43,44^ we aimed to provide a sufficiently detailed account of the data collection process, demonstrating transparency. Transparency is acknowledged in qualitative research standards as a more appropriate quality marker for the specific research design (i.e., interpretative description) and data analysis method (i.e., reflective thematic analysis) chosen for this study than data saturation.^45,46^

### Treatment Programs

The IIPT is comprised of a 3-to 6- week day hospital program (mean duration = 5 weeks). For 6 h daily, five days per week, youth and their families participated in goal-oriented rehabilitation therapies (i.e., physiotherapy, occupation therapy, psychology, recreation therapy, art and music therapy, academic support) aimed at enhancing their pain management skills, facilitating their emotional adjustment and coping, and improving their physical functioning. Alternatively, the outpatient MMT program includes a 1-day mandatory self-management pain education session for parents and youth and, once completed, individual physiotherapy (e.g., functional stretching, strengthen, postural reeducation, and endurance training through physical activity), psychology sessions (i.e., using a cognitive behavioral approach), medical treatment (e.g., medication regimen), and home programming are offered as clinically indicated. The aim of the MMT program is also to improve youths’ self-management abilities, emotional coping, and physical functioning, with minimal disruption to their regular lives. The duration and dose of the individual sessions and the need for other disciplines (e.g., family therapy, occupational therapy) are tailored for each participant with discharge contingent on the achievement of patient-identified goals. Both interventions are publicly funded in Canada.

Aligned with the family-centered care philosophy of the complex pain clinical service, once treatments were deemed appropriate, a team member (e.g., physician or nurse) presented the options to families, who were then asked to choose a treatment (i.e., either IIPT or MMT). Unlike other similar programs in North America, admission to IIPT is not contingent on the failure of MMT. Based on previously reported data, family treatment choices were based on clinical team recommendations and other family-based needs.^47^ Although some of interviewed participants in IIPT experienced the MMT prior to IIPT (*n* = 2), many families opted immediately for IIPT (*n* = 9).

### Procedures

Approval from the Conjoint Health Research Ethic Board, University of Calgary was obtained for all study procedures prior to initiating participant recruitment and data collection (Ethics #REB16-0916; 2017-1543). Once the consent process was complete, youth and parents were contacted to schedule an interview and provided with instructions on how to create a timeline of the child’s chronic pain journey, including their treatment experiences.

In clinical research, the collection of patient narratives can generate open-ended and inclusive stories, which may underscore unanticipated ideas and highlight previous unconsidered relationships, explanations, and solutions to clinical issues.^7^ These stories include a plotline and characters and are reflective of ongoing meaning-making associated with a certain condition, depending on the chosen events and their chronological order.^7^ Time is an important feature of the participants’ stories, defining and intrinsically weaving together an individual’s narratives, and helping to create meaning from the experience.^48^ Timelines are visual depictions of life history and events that can provide context and structure to narrative interviews and allow interviewees to reflect on their condition, their journey, and their experiences, using the temporal dimensions of the past, present, and future. They have also been reported as useful for data comparison by placing a clinical problem in the context of other salient life events.^49,50^ The timeline approach has been reported as empowering for participants, allowing them to take charge of framing their own realities.^48,51^ Moreover, it discourages researcher–research participant power imbalances that can exist and must be carefully managed, in particular when youth are involved.^48,51^ This data collection procedure was chosen in response to guidance provided by the study’s advisory committee. All committee members, including youth with chronic pain and their parents, agreed that understanding the relativity of the pain experience across time was important and timelines provided a methodological tool that could achieve this.

An extract of the timeline development instruction and semistructured interview schedule is provided in Table 2. As per previous research protocols,^48,49^ the time frame used was determined by the participants, with encouragement to focus on the period in their lives when pain (or their child’s pain) was a concern. The timelines ranged from 3 years to whole lifetimes (see example in Appendix A) and most were completed prior to the scheduled interview.

**Table 2.** Semi structured interview schedule

Interview Steps	Interviewer’s Script
General Directions	Please draw a timeline of your life up until now and mark the most important events and the changes that have happened as it related to your (or your child’s) pain journey.
Prompts	Please tell me what was happening in your life at this time *(interviewer points to an area on the timeline)*. Please tell me what was happening in your life immediately after the program *(interviewer points to an area on the timeline)*. Please tell me what was happening in your life a few months after the program *(interviewer points to an area on the timeline)*
Potential Sub-Prompts	What was happening at home/in your family? What was happening at school? What was happening with your friends/relationships? How were you feeling? How do you think your pain condition and its treatment impacted this?

To ensure appropriate interpretation of participants’ timelines, individual semistructured interviews were conducted with each participant by one coauthor (KH), who is experienced in qualitative interviewing in this setting.^18,52^ Parents and youth were informed that KH was not involved in the delivery of services with either program and that this work was associated with a doctoral dissertation. Using the timeline as a memory aid to facilitate the recollection, sequencing, and reflection of personal events, participants led the interview process, with KH simply identifying events on the graph by asking, “What happened here?” or “Tell me more about this.”^48^ Specific inquiries about school, family, peer relationships, and other meaningful activities (e.g., sports, recreation, work) were used occasionally (as per the interview schedule subprompts listed in Table 2). In-depth interviews are designed to elicit a vivid picture of the participant’s perspective on the research topic and an effective method for stimulating talk concerning a variety of topics, experiences, perspectives, personal feelings, and opinions, allowing insight into how people interpret, order, and create meaning in their own worlds.^53^

The data collection procedures, including the timeline creation and interview process, were pilot tested with the youth and parent advisory committee members guiding the larger study. The purposes of these pilots were to generate feedback on the technique and its relevance, identify the optimal time required for the interview, and test the appropriateness of the interview prompts. The feedback received was used to refine the procedure in the following ways. To facilitate inclusion, participants were provided with a range of locations where the interview could be conducted (i.e., their homes or a quiet room at the hospital) and methods were expanded from predominantly face-to-face interviews to include other information and communication technologies (i.e., telephone, FaceTime, Skype, and e-mail). Although many participants chose to participate in face-to-face interviews conducted in a quiet room at the hospital (*n* = 10), telephone (*n* = 9) and videoconference (i.e., FaceTime [*n* = 4] and Skype [*n* = 1]) were also employed. At the end of the interview, all participants were invited to e-mail the interviewer with any additional information. Three participants chose to supplement their interviews via an e-mail received within 2 days post-interview.

Participants were provided with the timeline instructions and the interview schedule prior to the interview to allow preparation and reflection and to decrease anxiety. At the time of the interview, youth and parents were reminded that the interviews would be audio-recorded and transcribed verbatim, and permission was obtained for interview quotes to be published. Interviews ranged in length from 25 to 150 min. Reflective memos and field notes were maintained by the interviewer (KH) throughout the interview process, with a particular focus on situational, relational, and performance reflexivity.^53^

### Data Analysis

Similar to previous studies using timelines, inductive reflexive thematic analysis guided our data analysis process.^38–41,54^ More specifically, the six phases described by Braun and Clarke^55^ and Braun et al.^56^ were used. Firstly, familiarization with the data was achieved through listening to the interviews and active reading and rereading of the transcripts. During this iterative familiarization process, initial codes were generated from data segments relating to the pain trajectory and the effects and impacts of either treatment program, following which they were organized into potential themes.

Guided by an analysis procedure previously used in a qualitative study involving youth and parents,^8^ all IIPT youth interviews were initially reviewed, followed by parental interviews for this same intervention. Themes were then compared across youth and parent transcribed interviews to identify patterns that were common and those that differed. Subsequently, the same analysis process was conducted for the MMT group. In keeping with the timeline representation, codes were initially grouped into time periods. Negative or positive categories emerged for data related to each intervention and were interwoven into the time continuum. The time periods and categories were then contrasted across treatment groups to identify intervention group patterns.

These initial coding and theming steps were completed by the first author (KH). Throughout this process, debriefing was conducted with CC, AB, and MN to discuss the development and interpretation of themes. Themes were named, reviewed, and refined, and concise definitions were generated for each theme with the assistance of CC, AB, MN, AJ, and JW. Agreement was achieved on the analysis and interpretations among coauthors, providing credibility and trustworthiness to the analysis data and interpretation processes. Themes were transformed into draft visual graphics, using a timeline as its foundation for reporting purposes. These graphic representations were reviewed by three interviewees (i.e., a youth MMT participant, a youth IIPT participant, and a parent, unrelated to these youth, who had experience with both the MMT and IIPT interventions) in a member-checking process to seek clarification and further explanation and ensure accurate representation of their experiences.^53^ In keeping with the inductive thematic processes, frequencies of themes were not counted, because the importance or meaningfulness of a theme does not necessarily equate with frequency or quantifiable measures.^56^ Throughout the analytical process, data were managed using QSR International’s NVivo^12,57^ a computer-assisted qualitative analysis software package, to check the validity of the translation into graphic representation for the whole sample. Attention was paid to including quotations from various participants for representation of all perspectives within each theme.

Numerous steps were taken to address the quality of this research study and its qualitative analyses. Aligned with good qualitative research methodological practice and to enhance the credibility of this study, the authors’ backgrounds, experience, and expertise in the field associated with this study and their specific role in the analysis are of relevance and are therefore briefly described.^53,58,59^ At the time of conducting the interviews and analysis, KH was completing a doctoral degree in health services research and had many years of rehabilitation experience with youth with disabilities, including those with pediatric chronic pain. AB, an established researcher, with expertise in program evaluation and qualitative methodology, oversaw the effect analysis, including the formulation of the evaluation question and review of the final analysis. JW, a researcher with qualitative methodology expertise, in particular timelines, provided guidance on the implementation of the timeline data collection tool, advised on the analysis, and reviewed the final analysis. AJ and MN, both researchers with many years' of experience working in the field of pediatric chronic pain and with expertise in the use of qualitative and narrative methods, provided advice on the data collection phase, reviewed initial timelines for content, helped guide the data analysis, and offered content expertise on the themes and final analysis. NR, a clinical expert in pediatric chronic pain and with specialized experience in multidisciplinary pain rehabilitation, also provided content expertise and reviewed the final themes. Finally, CC, who has many years of experience in pediatric rehabilitation as a clinician, evaluator, and researcher and expertise in participatory and qualitative methodological and a variety of analytical approaches, provided advice during the data collection, reviewed the initial coding of the interviews and thematic scheme, and offered guidance on the final themes and their presentation. The use of both drawings and narratives further enhanced the credibility as the researchers triangulated the results across both mediums.^60^ A member reflection process, a technique of discussing findings with participants, providing opportunities to question, critique, and provide feedback, was also used.^61^ Finally, trustworthiness was ensured through the presentation of quotations from across a varied range of our participants’ accounts.^59^

## Results

Participants’ narratives were extremely complex, spanning many years, describing linkages between short-, medium-, and long-term treatment outcomes. The themes generated for each of the interventions included (1) struggling to find a cause, a cure, and to keep up; (2) acquisitions and disruptions; (3) supports and realities; and (4) pain and life and were associated with a specific time period, relative to the youth’s pain and treatment journey.

**Figure 2.**
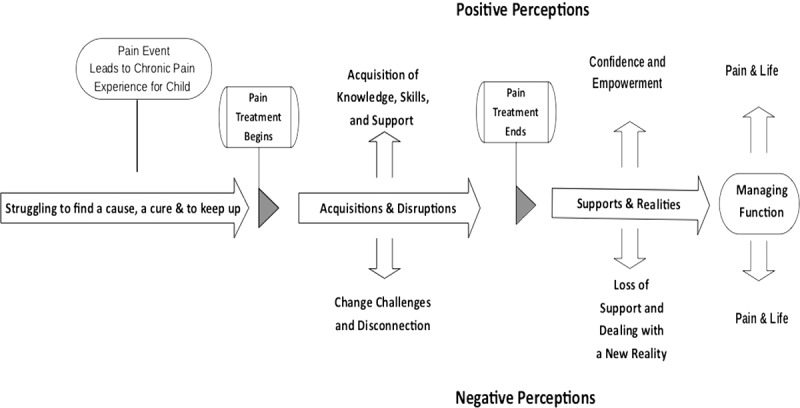
IIPT youth and parent participant perceptions of treatment. Final themes and subthemes generated from youth and parent IIPT participants’ interview transcripts across their pain and treatment trajectories

**Figure 3.**
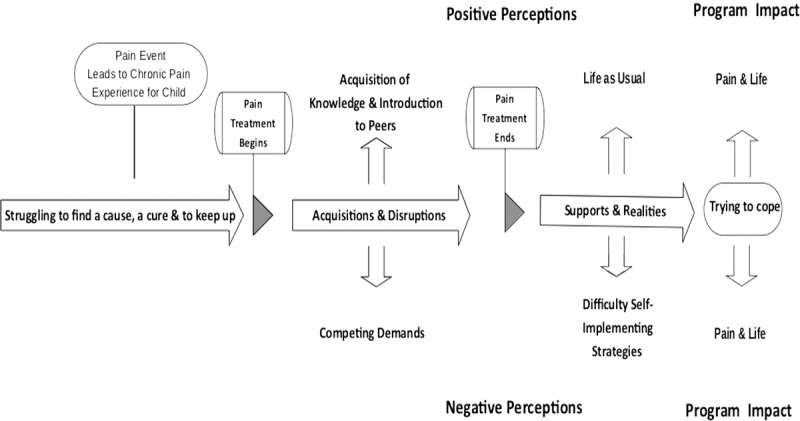
MMT youth and parent participants’ perception of treatment. Final themes and subthemes generated from youth and parent MMT participants’ interview transcripts across their pain and treatment trajectories

Struggling to find a cause, cure, and to keep up related to the period before accessing specialized multidisciplinary pain rehabilitation services and was reflective of the consequences of the pain experience itself. Acquisitions and disruptions occurred during treatment, ending at discharge, and were associated with the immediate effects of participating in the treatment. Supports and realties were linked to medium-term outcomes surfacing months and even years following the intervention. Pain and life related to participants’ current status, reflecting the longer-term impacts perceived to be a result of participating in one of the two programs. Figures 2 and 3 provide a graphic representation of all four themes, when the treatment occurred, and the relationship with the timing of the intervention.

As illustrated, the aforementioned four main themes crossed both treatment options, and the generated subthemes were specific to each program. The themes and subthemes for each intervention are further compared in Table 3, where the themes and their subthemes along with a list of the codes are presented. Detailed descriptions of each of the themes and subthemes, accompanied by representative youth and parent quotations illustrating their perspectives and experiences, are provided in the following subsections. Additionally, the subthemes identifying positive and negative effects of treatment options and the final perceived program outcomes will be further contrasted. To protect participants confidentiality, pseudonyms have been used.

**Table 3.** Comparison between the IIPT and MMT themes and subthemes

Time	Theme		
Before specialized services	Struggling to find a cause, a cure & to keep up	*Codes* 1. Increasing school and work absences 2. Loss of interest in hobbies and cessation of sports and physical activities 3. Loss of friends 4. Missing family outings and vacations 5. Lack of enthusiasm for school and social activities 6. Social isolation 7. Increased dependence on parents 8. Parental and family stress 9. Emergence of depressive symptoms 10. Initiation of negative pain coping cycle 11. Constant advocacy for specialized pain services	
During treatment	Acquisitions & Disruptions	**IIPT** **Positive — Acquisition of Knowledge, Skills, and Support** *Codes* 1. Meeting and daily interactions with peers and parents with similar challenges and experiences 2. Acquisition and integration of pain knowledge 3. Repeated practice of various pain strategies in a supportive milieu with readily available coaching 4. Parental training in coaching and support **Negative — Change Challenges & Disconnection** *Codes* 1. Physical and emotional exhaustion 2. Challenging to parent–child relationship 3. Absence from school and work 4. Distance and accommodation away from home 5. Missing family events 6. Communication challenges between parent unit 7. Challenges in meeting sibling needs	**MMT** **Positive — Acquisition of Knowledge and Introduction to Peers** *Codes* 1. Acquisition of preliminary pain knowledge and introduction to management strategies 2. Introduction to other peers and parents with similar challenges and experiences 3. Readjustment of parental expectations of their child **Negative — Competing Demands** *Codes* 1. Frequency of school and work absence 2. Strain on youth and parent due to travel associated to and from multiple appointment 3. Difficulty access recommended services in some communities 4. Difficulty problem solving and practicing pain strategies between appointments without close supervision
Following Program	Supports & Realities	**IIPT** **Positive — Confidence and Empowerment** *Codes* 1. Confidence in knowledge and skills and confidence to self-manage 2. Empowerment in ability to attend school consistently and improved school attendance 3. Program legitimizes pain to school personnel and facilitates negotiation of accommodations 4. Emotional support from and friendships with program peers 5. New peer networks and improved socialization skills 6. Discovery of new leisure and sport interests **Negative — Loss of Support and Facing a New Reality** *Codes* 1. Lack of clinical support following program 2. Back-to-school stress and impact on postsecondary plans 3. Loss of previous support networks and daily connections with pain peers 4. Consequences of uncovering family issues and being unable to resolve them within the program time frame 5. Loss of rigorous structure postprogram and therefore some associated gains 6. Recognition of the lifelong permanence of pain condition	**MMT** **Positive — Life as Usual** *Codes* 1. Application of knowledge 2. Minimal disruptions to routine 3. Remain with peers and in community school **Negative — Difficulty Self-Implementing Strategies** *Codes* 1. Youth require parental support to self-manage 2. Frequent school absences due to pain 3. Continuation of negative pain coping strategies 4. Socialization only when pain and fatigue allow
Impact	Pain and Life	**IIPT** **Managing function** *Codes* 1. Living with pain, focus on function and gaining control 2. Doing what ones wants to do, needs to do, is expected to do 3. Resumption of age-appropriate roles and responsibilities 4. Prioritization and choices 5. Pain in the background	**MMT** **Trying to cope** *Codes* 1. Focus still on pain and symptom reduction 2. Pain remains a limiting factor to function, roles, and responsibilities 3. Pain in the forefront and a heavy burden

### Theme 1. Struggling to Find a Cause, a Cure, and to Keep Up

Struggling to find a cause, a cure, and to keep up captures the crux of the participants' narratives related to the initial negative consequences of the youths’ pain journeys. For most, the pain journey began with a specific event when the pain first emerged (e.g., injury, illness), whereas for others, it was marked by a time point (e.g., a specific date) or a life event (e.g., child’s school grade, parent employment). For both intervention groups, participants described an increase in pain intensity, locations, or frequency between the emergence of the pain sensation; fear that an important health condition was being missed; and frustration in gaining access to specialized services. The lack of answers received from health care professionals increased youth and parental frustrations, which was further exacerbated by youths’ deteriorating function. This participant recounts her experience:

I was on a hike and I started to feel this pain. [The pain] kept increasing throughout the summer. We started to get it checked. I received physio, chiro, active release, and then I started getting x-rays and CAT [computed tomography] scan. They could not find anything. I was getting super frustrated, but we kept going. (Adelaide, 17 years, MMT youth)

As symptoms persisted over time, these frustrations remained, regardless of whether or not a diagnostic label was provided, and was evidenced in both groups.

Furthermore, youth and parents battled to continue with the demands and expectation of everyday life, while being progressively burdened by the deteriorations caused by the pain. Both parent and youth participants’ narratives expressed a spiraling loss of function in all areas of life as time progressed. In addition to declines in physical function, they also portrayed deteriorations in youths’ psychological well-being, family roles, and school and social engagement. This participant described her experiences:

We went on a [family] trip to Florida. I could barely stand in line for the rides and I didn’t have any fun. When I started a new school, I had a really hard time socializing and missed a lot of classes, because I was having pain. I started to be afraid to go to school. I wasn’t sleeping at all. (Olivia, 17 years, IIPT youth)

Parents also provided vivid accounts of their desperation during this time, which spoke not only to the impact on their child but to how the pain affected the immediate household, the broader family, and their community. This participant provided this powerful exemplar of the consequences of her daughter’s chronic pain:

[My daughter] was having three-hour panic attacks every night. I was massaging her, talking to her and really not having any skills and not knowing what I was doing. My anxiety was through the roof for two years. Of course, that affected the others [family members]. (Rose, IIPT parent)

The theme struggling to find a cause, a cure, and to keep up provides the contextual background for the other themes and their associated subthemes. In describing their perceptions of participating in treatment and the effects and outcomes overtime, youth and parents often made reference to this pretreatment time period as a means of underscoring the relevance of their statements, of giving meaning to the pain journey, and of justifying their treatment choices.

### Theme 2. Acquisitions and Disruptions

The theme acquisitions and disruptions depicts the effects of participating in specialized multidisciplinary pain rehabilitation interventions. These immediate effects were related to many implicit and explicit factors, including youth and family intervention expectations, the intervention itself, and family circumstances. Many youth, although cautiously optimistic, also expressed self-protective attitudes and a reluctance to pursue specialized multidisciplinary pain interventions because of previous disappointing or failed treatment experiences. One participant described her situation:

It was mostly my dad because I had missed so much school and he was very concerned. I wasn’t really sure about it, because at this point, it had been years trying different things, and I was not 100% convinced that this would help. (Danielle, 18 years, IIPT youth)

The reasons for choosing the specialized pain treatment, youth attitudes toward the intervention, their involvement in the decision-making process, and parent and youth treatment  expectations influenced participants’ perceptions of treatment participation and its effects, in particular whether a positive or negative label was assigned. Positive and negative effects were identified in both treatment options (i.e., IIPT and MMT), some similar, others quite different. In presenting these effects, comparative details will be added as appropriate.

#### IIPT Positive Effects: Acquisition of Knowledge, Skills, and Support

The acquisition of knowledge, skills, and support subtheme comprised the most commonly cited positive effects and valued attributes of participanting in the IIPT. Gaining knowledge and having the opportunity to apply it to daily life situations were perceived as the cornerstones and most valued activities of IIPT. More specifically, participants articulated the importance of pain education during the program in improving their understanding of pain (e.g., mechanisms, triggers) and the strategies to manage it. This participant explained her feelings:

At the end of it, it felt like so much had changed. I didn’t have a lot of pain anymore and when I had it, I had an understanding of why and how I could manage it. (Dominque, 19 years, IIPT youth)

Parents also acknowledged the benefits of sharing the same knowledge as their child, which included a better understanding of the pain and the strategies their child had been taught. Additionally, many parents reported gaining an awareness of the negative contribution of their own behaviors on their child’s pain and recognized a need to change their parenting style and to acquire different skills. The positive effect of the simultaneous acquisition of pain knowledge and skills by parents and youth was vital in changing parenting behaviors and enhancing youths’ ability to cope with their pain. As this participant explains, the knowledge and skills acquired changed the communication within the parent–youth relationship, creating a new language to use between them, as well as with the families and their community members:

Understanding chronic pain, learning about the beast that it actually is, and what it looks like in the future, learning how to communicate about it, was a huge help. What it offered our family was the language to understand, communicate, about, to learn to accept the pain. (Rose, IIPT parent)

The supportive milieu created during IIPT was also viewed as a positive effect of participating in this intervention. Peers and staff support were both acknowledged as beneficial. Parents and youth recalled meeting others facing similar challenges with much fondness. Phrases such as “no longer feeling alone or isolated” and “feeling understood” permeated their discourse. As captured in this participant quotation, many also highlighted a sense of feeling accepted and not judged in this peer context:

[Meeting other parents] is very therapeutic. You talk about certain things you can’t talk about with other parents. Even though we have group therapy, where the parents get together, it was based on clinically scripted questions. When you are just chatting, there are things we are able to say that we might not in a group setting. There is a comfort with what we can say, being able say it, and not be judged. (Alice, IIPT parent)

Policies limiting contact and communication between IIPT participants during treatment were criticized by most youth and parents. Other benefits attributed to participating in the IIPT peer group programming included increased motivation to challenge oneself through friendly competition, the development of empathy, the recognition of others’ struggles and a focus on successes, as well as shared learning.

In addition to peers, the support of IIPT staff was identified as a positive effect of participating in this program. Many participants described vivid examples of clinicians providing youth with support during challenging times (e.g., pain flares) and modeling appropriate coaching behaviors for parents, which were perceived as vital to both parent and youth skill acquisition. One mother described how it transpired for her:

I was hesitant because I didn’t know medically how far to push [my daughter]. [The physio] knew how to push [my daughter], through. I didn’t know if I could do it without hurting her. Witnessing [the staff] being able to push [my daughter] through things was helpful. The next pain flare that she had, I just talked her through it. And she was able to use some of the strategies she had learned, and she got herself through a really bad spell. She just needed a little bit of coaching from me. (Alice, IIPT parent)

#### IIPT Negative Effects: Change Challenges and Disconnection

The IIPT negative effects subtheme, change challenges and disconnection, depicted the extreme effort required to change behaviors and the sacrifices required to do so. The program intensity and the physical and emotional effects required to participate were the most commonly cited challenges. Youth and parents both recognized the intense, time-limited structure and the very high participation expectations as incredibly difficult to manage. This participant provided this vivid memory of completing IIPT:

I was completely exhausted at the end of the [program]. They just worked us so hard. It was mentally exhausting, physically exhausting, emotionally exhausting. So, I would say that the immediate effects were kind of negative. (Mila, 19 years, IIPT youth)

Some IIPT parent and youth participants also ascribed negative effects related to the disconnection from their regular lives and daily routines required to participate in the IIPT option. For those who lived at a significant geographical distance, the burden of being away from their partners, siblings, other family members, in addition to missing important events and celebrations, was perceived by most parents and some youth as negative effects associated with treatment participation. More commonly, however, youth highlighted the loss of regular connection with their peers and academic milieu. As expressed by this participant:

I found so much support at school with my friends, so it was really hard to be away from my support system during the program. (Luisa, 18 years, IIPT youth)

Some reported making efforts to maintain contact with friends on weekends, and others used technology (e.g., group video games), social media, and text messaging in an attempt to remain socially connected.

In addition to family and social disconnection, being away from school for the intensive period of time required for IIPT engagement was perceived as one of the most significant burdens recognized by participants. Factors such as the timing of the program, participant’s grade level, prior school attendance, and youth’s involvement in making the decision to participate in the IIPT influenced youth perceptions. This participant described her struggles:

I definitely found it difficult. I was taking a full course load during the program and, because I was taking grade 12 courses, if you’re not there, you have to do double or triple the work to catch up. (Danielle, 18 years, IIPT youth)

The negative effects of treatment participation on parents’ lives were also underscored. Loss of productivity associated with work, communication challenges, and compromises associated with parental duties were commonly cited challenges. All IIPT parent participants highlighted that at least one parent within their household worked part-time, was the full-time family caregiver, or owned their own business. This flexibility was acknowledged as being key in affording these parents and their child the opportunity to participate in the IIPT. Furthermore, empathy was shown toward families for whom these arrangements were impossible and for whom a significant participation burden was perceived (e.g., distance from home, single parent).

#### MMT Positive Effects Subtheme: acquisition of Knowledge and Introduction to Peers

Acquisition of knowledge and introduction to peers was selected as the subtheme title for the positive effects of participating in MMT because it encompassed the most valued activities identified by these participants. In contrast to IIPT, most MMT parents described the full one-day session in which pain education was provided to parents and youth separately. Meeting peers in these session was important in validating youths’ pain experiences and in fostering resilience. Furthermore, parent participants identified key messages that they integrated during the sessions, as well as those that resonated with their child. This participant provided this example:

[My daughter] met a whole bunch of other kids who are living with pain the way she is. I think it helped to know she’s not alone. But she also realized living with pain is tough and that sometimes you just have to suck it up and push through. (Elena, MMT parent)

Similar to IIPT, parent participants also acknowledged the benefit of meeting other parents “that are trying to navigate what it looks like to be a parent of someone in pain” (Elena, MMT parent). Furthermore, they underscored the benefits of learning the same information and strategies as their child and gaining an awareness of realistic, developmentally appropriate expectations to have of their child, despite their pain condition. Their queries ranged from school attendance, to social engagement, to family roles. One parent expressed her learning this way:

Still expecting him to do chores. They said, “You need to stop doing everything for him and make him do some of it himself.” Because he has to learn to live with this pain. That I didn’t think of and I know the other parents there didn’t think of it either. So then, [my son] and I had a talk and I said, “OK, I learned this; you learned that, this is what we are going to do.” That helped me a lot. (Delphine, MMT parent)

As exemplified in this quotation, much like the IIPT participants, the MMT parents also underscored an awareness of the need to change overprotective and solicitous parenting responses to their child’s pain. Furthermore, they also acknowledged the pivotal role of the pain education in creating a common language and understanding between them and their child.

#### MMT Negative Effects: competing Demands

The subtheme competing demands designates the negative effects of MMT, portraying the clear tensions reported by youth and parents in their attempt to adhere to treatment recommendations while trying to meet regular life expectations. Unlike IIPT where youth and parents were disconnected from their lives for a specific period, for MMT youth and parents, treatment recommendations required inclusion in their already very full and busy lives. Weekly appointments and the associated travel as well as difficulty accessing suggested services when and where they were most needed were commonly reported challenges. Appointments and the associated travel time were identified as important impediments to school and work attendance and productivity. For some families, the distance they lived from pediatric specialized pain rehabilitation services and the lack of specialized services (e.g., pediatric psychology) in their communities created additional challenges and dilemmas, as explained by this participant:

There were recommendations from [the team] and I just didn’t know where to go and who were experts in dealing with children. I didn’t want an adult specialist counseling my 13-year-old. I want someone who specializes in adolescents and that doesn’t exist in [my community]. Due to work, there were times when I had to rely on my parents to make a few trips up to [the city] for me. It was difficult but we did our best. (Fleur, MMT parent)

Although distance was also mentioned as a challenge for IIPT participants, in their case it was related to being separated from loved ones for a period of time and less about the repeated distance traveled and associated productivity losses (i.e., work and school time). Furthermore, lack of access and availability of local specialized multidisciplinary pain rehabilitation services was identified by some families as a precipitating factor in opting for IIPT. Finally, many youth participants in MMT also discussed the difficulties encountered in applying pain management knowledge and strategies into their everyday lives, as this participant expressed:

They offered me physio and then psychology, every 2 weeks, back-to-back. I would see them both in the same day so I wouldn’t have to travel multiple times. However, I would learn a strategy and then would often have to wait until I did something active to practice the strategy again and then, say, if I had pain or if I was struggling, I would have to wait for two weeks before having help again. (Sabrina, 17 years, MMT youth)

In contrast, this lack of practice and access to clinical support when needed was not raised in IIPT. Instead, the ability to practice learned skills and receive timely feedback was underscored as a positive effect of participating in the IIPT option. These positive and negative effects of treatment participation often influenced youth and parent participant perceptions of the longer-term outcomes and impacts.

### Theme 3. Supports and Realities

The theme supports and realities defined the more sustained, profound, and longer-term program outcomes as perceived by those enrolled in pediatric specialized multidisciplinary pain rehabilitation. Their description often referred to the consequences of youths’ chronic pain prior to accessing specialized multidisciplinary pain rehabilitation and typically built on the effects of their participation in treatment. This is evidenced in the description by this participant:

I can completely handle my pain now even though I still have it sometimes. I have a physical and mental strategy to completely get [my pain] under control. Up until the program, [my pain] was essentially like a massive obstacle that was unscalable. Now, if pain is becoming an issue, I can deal with it. I also had social support coming from the IIPT. It gave me like my first toolkit of adult social skills. (Adrian, 19 years, IIPT youth)

These sustained program effects were often explained in relation to intervention goals; participants’ interpretations of their life situations, including their pain status; and the milestones they had attained and still hoped to achieve. These relationships influenced whether a positive or negative identifier was assigned to the program outcomes.

#### IIPT Positive Outcomes: Confidence and Empowerment

The subtheme confidence and empowerment describes the positive impact highlighted by youth and parent IIPT participants of the belief in themselves and their ability to manage the pain and subsequently control their lives. Gaining confidence in self-management was described as the most positive outcome of the IIPT program. Participants described achieving this confidence by acquiring the required knowledge and skills, a recognized positive effect of treatment participation, and essential in achieving self-management. One participant explained the changes he observed in his son:

I think the program changed him because he got a lot of confidence and I think he understood the nature of his pain and how it was affecting him. And above all, what he could do about it. (Jean, IIPT parent)

For youth, experiencing success in the application of the knowledge and skills to various contexts and problem solving through challenging, atypical, complex, or unplanned situations was particularly helpful in creating this self-reliant belief. Some IIPT participants reported having setbacks in pain management and relapses since their discharge from the program. Setbacks were associated with a series of emotionally difficult life events (e.g., death of a family member, repeat injury). However, despite these, youth, along with their parents, expressed self-confidence in their abilities to return to self-management. This self-confidence was also fueled by their sustained peer support network created during the IIPT. Many youths and some parents reported having maintained contact with other IIPT participants. They highlighted that these relationships were founded on not only shared pain experiences but also interests outside of pain (e.g., shared education goals). Moreover, youth credited their IIPT peer support network with enhancing their coping skills and helping maintain their physical and psychological well-being following the treatment, even months and years later. As one participant shared:

Following the [program], I had a depressive relapse. But I had two close friends from the program, and so I had social support. It was a really small episode; much shorter than others I had had. It gave me confidence to know if something goes wrong, I still have people that I can fall back on, besides my family. (Adrian, 19 years, IIPT youth)

The enhanced social skills and reestablishment of meaningful peer relationships following IIPT were also attributed to the development of social networks during treatment. In addition to social skills, many participants associated their new peer networks with new interests, hobbies, and meaningful activities, first experienced during the IIPT. This participant provided this explanation:

A huge challenge for [my daughter] was to connect with people who weren’t active and involved in [her sport], but she still found interesting. One thing she discovered through the program was music. She was able to connect with other friends in her love of music that allowed her to find other ways to connect. (Sophia, IIPT parent)

Youths’ enriched social capacities empowered them to develop and refine their self-advocacy skills. More particularly, this applied to advocating for modifications and accommodations in school, drawing on supports they had received during treatment. As one participant described:

Accommodations have allowed me to, if it’s a written exam, I can type it. Writing that much by hand is too much for me. I requested [the accommodation] actually coming into university because I knew that I relied heavily on them in high school. One of the doctors in the program filled out the form for me that the university required, and the people at the accessibility centre were very understanding. (Luisa, 18 years, IIPT youth)

As highlighted in Luisa’s narrative, support was required to accompany these requests. Many youth acknowledged that participation in IIPT was enough to validate their condition to the outside world, specifically to people who were skeptical about the existence of their pain.

#### IIPT Negative Outcomes: Loss of Support and Facing a New Reality

The subtheme loss of support and facing a new reality described participants’ negative experiences and feelings associated with leaving the protective, nurturing IIPT milieu and transitioning back to the expectations of everyday life. Many parents and youth acknowledged the significant struggles of this transition. As expressed by this participant:

[My daughter] came from alienation, having an invisible condition and people not believing her, to this beautiful little bubble, the program, where she was validated, supported, and encouraged, and then dropped back into the real world. I know that part of the program is to teach you to live life independently with your pain. But she was riding a high, ready to conquer the world, with nothing but supportive people around her. And then there was nothing. It was a bit tough. (Alice, IIPT parent)

Moreover, some participants expressed anger toward the program staff, sharing feelings of perceived abandonment, in particular if issues identified during IIPT (e.g., family conflict) were perceived to be unresolved prior to discharge.

Negative program impacts were also associated with the repercussions on school and social engagement. IIPT youth narratives were peppered with multiple examples of the challenges they faced when returning to school following treatment. These included feeling forgotten by classmates and undervalued by teachers and the excessive and overwhelming academic catch-up required to meet academic performance targets (e.g., course credits). Some of these impacts had long-term negative impacts on youths’ postsecondary academic paths and the achievement of their ideal careers, as exemplified by one participant:

I had a really good average coming out of high school, but those two classes I took during the IIPT set my average back. I really wanted to get into the Neuroscience Program. But when I got the marks back from two exams, I wrote during the IIPT, they were quite a bit lower. I didn’t get into neuroscience and instead settled for biology. (Luisa, 18 years, IIPT youth)

In addition to highlighting the negative outcomes on school engagement, some participants discussed the negative impact on peer relationships. For example, some youth noted that new peer groups had formed during their absence and private jokes had emerged that they failed to understand. These situations were often referenced in relation to the amount of time spent away from their peer group and had the unfortunate effect of leaving many with feelings of exclusion, isolation, and alienation all over again. These negative program impacts were often not evident in the discourse of MMT participants, and the positive program impacts varied widely.

#### MMT Positive Outcomes: Life as Usual

The subtheme life as usual highlighted MMT participants’ reflections on the minimal perceived disruptions caused by the treatment to their regular lives. Most participants normalized the daily accommodations made for pain in their descriptions of their routines. Their descriptions focused on their lives within their communities, including school, peers, and family life, and were less focused on the hospital services received compared to those in IIPT. Parents and youth provided examples of the positive outcomes of MMT. More specifically, it allowed them to create partnerships and alliances with local school personnel that were perceived as vital in creating an academic plan that worked for the youth. Most parent narratives identified a member of the school personnel (e.g., principal, guidance counselor, teacher) who was instrumental in facilitating an individualized plan and who had taken the time to get to know the child, their condition, and their academic capabilities. Furthermore, the use of a combination of learning methods, in particular online courses, was more common in this intervention group than in IIPT. This participant provides this description:

I just really can’t get up at the correct time to get to school in the morning because of my pain. I have to sleep a little bit longer and I can because I do some online schooling instead of morning classes. So, I do a lot of my schooling at night instead. I have one class at [the community school]. It’s the last class of the day and is my option class. (Brittany, 17 years, MMT youth)

Similar to IIPT, the knowledge acquired in participating in the MMT program was applied by youth in identifying and proposing accommodations to help them in school. However, unlike the self-advocacy noted in the IIPT youth participants, parents often negotiated the accommodations with the school administration instead of the youth themselves.

The maintenance of peer relationships in and outside of school was also noted to be a positive impact of the MMT program. Despite highlighting similar peer-related issues (e.g., bullying, teasing) as those enrolled in IIPT, most MMT youth participants made references to long-standing friendships, with either one individual or a small group of peers. This participant described her friends like this:

I always had close friends, actually a close group of friends. The therapists I saw suggested that strengthen my entire leg would help my foot. My friend also just wanted to start something new, so she came with me. We often go to a gym together and have a personal trainer now. (Adelaide, 17 years, MMT youth)

#### MMT Negative Outcomes: Difficulty Self-Implementing Strategies

Difficulty self-implementing strategies describes youth limited ability to integrate pain strategies into their daily lives and was perceived as a negative outcome of the MMT intervention. In comparison to the IIPT program, MMT participants’ pain knowledge was more superficial and more parental input was required to assist youth in managing their pain. For example, some participants had difficulty naming triggers that exacerbated their pain, as demonstrated in this example:

My mom thinks that when I’m stressed [my pain] gets worse. I have no idea. (Adelaide, 17 years, MMT youth)

Furthermore, examples of effective implementation of management strategies to foster function were rare and evidence of negative pain coping strategies remained. The lack of effective pain coping strategies negatively impacted school attendance and social engagement, as highlighted in this participant’s description:

Last semester [my daughter] missed 51 days of school. When she has a pain spike, she cannot get out of bed. She had to drop out of a bunch of classes last semester. She’s now taking online courses. (Elena, MMT parent)

This participant provides insight into her limited ability to socially engage due to ineffective implementation of pain strategies.

[My pain] can keep me away from people sometimes, and sometimes I have to bail on plans. I’ve got some very close friends that I keep very dear to me and they understand that sometimes I can’t keep a plan and they get that. So, we just reschedule. (Brittany, 17 years, MMT youth)

Many other examples of youths’ reliance on their parents for pain management were reported.

### Theme 4. Pain and Life

The theme pain and life represented the perceived final impact of pediatric specialized multidisciplinary program options. These end results were quite different between treatment options and therefore will be presented separately.

#### IIPT Final Impact: Managing Function

Managing function was identified as the final impact subtheme for IIPT within the pain and life theme. It depicted the focus of IIPT youth and parent participants on their perceived function as a result of the program and their shift away from symptom reduction to one of living well despite their pain. When making reference to their function, participants included examples of what youth needed to do, were expected to do, and wanted to do. For many youths, although some level of pain or intermittent discomfort remained, it was rarely mentioned in describing their current daily lives. Youth self-management skills went beyond managing their pain to include gaining control over their pain and enabling them to fulfill most age-appropriate expectations, roles, and responsibilities. These expectations included family responsibilities, academic obligations, and social commitments. Compromises and prioritization of demands were often required to achieve the level of function needed. Despite their ability to manage their function, the complexities of chronic pain and its consequences remained in the background. This participant stated:

Because of the IIPT, [my daughter] is a high-functioning kid with pain. But every day she doesn’t feel good and every day is hard, and it’s a slog. (Rose, IIPT parent)

#### MMT Final Impact: Trying to Cope

The subtheme trying to cope was selected as the final impact for the MMT option because it represented the influence that pain still had on participants’ day-to-day lives, their ongoing focus on symptom reduction, and the acknowledgment of attempting to regain control. In providing descriptions of their day, many participants emphasized pain as the limiting factor in fulfilling family, academic, and social expectations, roles, and responsibilities. Furthermore, pain was more often referenced in their narratives. For most MMT participants, pain took center stage in their lives, still exerting much control over how they navigated through each day. As one participant stated “I just have to push through and get done what I want to get done” (Adelaide, 17 years, MMT youth).

As evident in this quote, pain remained a heavy burden, an obstacle to overcome to live life.

## Discussion

The purpose of this study was to explore and compare the effects of specialized multidisciplinary rehabilitation programs as perceived by youth participants and their parents using a narrative temporal approach. Some of our study results confirm existing research findings such as pain consequences reach far beyond the youth themselves, affecting parents, families, and peers^9,10,20,21,36^ and that functional losses occur in all life domains, including worsening physical and emotional well-being and health-related quality of life.^9–11,20,21,34–37,62^ Furthermore, many themes associate with diagnostic uncertainty—that is, haunted by something missing, the search for alternate diagnosis, and mistrust in the medical system described in previous literature^8^—were congruent with the discourses of participants across treatment groups prior to treatment commencement. More specifically, participants’ (i.e., youth and parents) prior pain and treatment journeys established a narrative about the truth and reality associated with the pain. These narratives integrally impacted treatment engagement, as well as how and whether participants benefited from their chosen treatment option and what benefits they perceived. How specific experiences prior to treatment commencement specifically influence youths’ trajectories during and after the program warrants more in-depth exploration.

Our study also underscored the unique positive and negative perceptions attributed to the two treatment options, as well as valued treatment components within each option. Acquiring knowledge and interacting with peers facing similar chronic pain consequences were the primary benefits common to participants in both programs. Moreover, they were viewed as instrumental in gaining the ability to managing the pain, for both youth and their parents. Although the benefits of pain education have been acknowledged in the adult pain literature,^63^ evidence in the pediatric population is nascent.^15,19,64,65^ The effects of pain education may be better understood when associated with peer interactions,^19^ also recognized as a positive treatment effect in our study. Group treatment reportedly creates an environment for normalization, for sharing experiences, and for reflecting on one’s own circumstances in contrast to others.^19^ However, concerns have previously been raised about the potential of peer interactions to contribute to youth further identifying with the sick role, fostering relationships founded solely on health and pain issues, leading to peer contagion.^66,67^ Along with Forgeron and colleagues,^68^ our findings refute these claims, demonstrating that peer relationships are not necessarily sustained on the common pain experiences alone but also resulted in the sharing of common interests outside of it. Researchers have described a curative aspect of connecting with peers, feeling understood for the first time, and a sense of validation and belonging.^20^ Our study participants credited the treatment milieu and its effects as enabling knowledge acquisition and skill mastery, reducing feelings of isolation, and enhancing coping and self-management skills.

Parents and youth participants also reported confidence and empowerment, pivotal to self-management, as the most common program outcome of IIPT. In addition to restoring parents’ confidence in their parenting, even in the presence of persistent youth pain, IIPT parents felt empowered as a result of gaining the knowledge and skills to help their child cope with the pain, a finding supported by previous evidence.^21^ The IIPT youth participants in our study also reported enhanced belief in their capacity to better manage their pain and to have more control over their lives. Furthermore, they expressed a renewed sense of hope and confidence associated with greater self-efficacy at discharge. Similar findings were reported in a previous qualitative study, where six youth expressed a renewed sense of hope, improved confidence, and self-efficacy at IIPT discharge.^20^ The IIPT participants in our study provided vivid examples of how this had been accomplished since the program’s end. On the other hand, MMT participants did not express the same level of commitment to self-management or degree of self-efficacy. This may be linked to adherence difficulties associated with MMT programs, as expressed by our MMT participants and reported by other authors.^69^

Perhaps even more important than the positive perspectives, our study findings also underscored some negative effects, outcomes, and impacts specific to each treatment option, which have previously been underexplored. IIPT participants felt disconnected from their social and academic communities because of the lack of daily interactions with their networks during treatment and communities during treatment. This is an important consideration when recommending IIPT to potential participants, because these networks are deemed critical to this age group.^70,71^ Moreover, maintaining friendships may be a way for youth with chronic pain to preserve their identity.^9^ In addition, disconnection from school, an important context for academic, cognitive identity, independence, and social relationship development,^72^ was often reported as a negative treatment effect by our IIPT participants but also by MMT participants experiencing frequent pain episodes and treatment appointments. Youth in both interventions raise education concerns and academic achievement worries. As a result, and as recognized by other researchers, these concerns place this group at risk of dropping out of school, exacerbating self-esteem issues and possible role loss within society.^9^ However, our study confirmed that sharing relevant information to receive the necessary modifications and accommodations from the school administrators and teachers can result in better reintegration of these youth in their roles as students, peers, and members of society.^9^ In highlighting their academic disconnection, some IIPT youth participants in our study also underscored the perceived negative outcomes of treatment on their school performance and its contribution to altering postsecondary plans and career paths. This finding is consistent with the reported perception that youth with chronic pain feel that they lag behind their peers in school progress and employment,^73^ even if some studies suggest that they are on track developmentally on milestones such as school graduation, college attendance, and independent living.^74^ Further research with older adolescents and young adults is needed to examine educational and vocational outcomes related to intervention options, because of its significant impact on the future socioeconomic status and financial independence of these youth.

Finally, many IIPT youth participants and their parents experienced challenges transitioning back to daily life after the program. The loss of support from program peers and staff was described as abandonment and detrimental as youth and parents struggled to adapt their knowledge and skills to real-life situations without support. Discharge planning and preparation for the transition back to their community have been suggested in the literature as important gaps and underscored as a need that requires further focus.^75^ Exploring the role of comorbidities such as anxiety, depression, or posttraumatic stress disorder following program completion also deserves more attention, for which potentially additional or even different interventions may be required.^76^ Booster sessions 3 months after IIPT admission may also be important to sustain improvements.^77^

A strength of this study lies in the exploration of both youth and their parents’ perspectives, given their unique needs. The qualitative nature not only provided an opportunity to better understand the processes of change and effects of two specialized multidisciplinary pain rehabilitation options but also offered important clinical information on how to improve care and the program components most valued by participants. The innovative methodology, using timelining followed by in-depth interviews, created rich temporal narratives because of the reflective preparation (i.e., producing the timeline) that preceded questioning. Moreover, it placed the participants in control of the process, allowing their stories to be told and anxiety to be decreased through the precirculation of interview questions. It also provided the opportunity to document both positive and negative intervention perceptions, across a time continuum, assisting in creating a trajectory of effects unexplored in previous effect analysis studies.

An important limitation of this study was its small sample size, in particular youth participants in the MMT. As a result, it may not be representative of the diverse experiences of those participating in this intervention. Furthermore, because of the optional nature of participation in the study, it is possible that our results may be slanted more toward those who experienced a positive engagement with the health care team and treatment. In an attempt to mitigate these limitations, a conscious effort was made to examine deviant cases as a means of capturing broader variations of perceptions on pain and treatment journeys, expanding the breadth of the sample, despite its size. The small sample did allow for more in-depth interviews. The diversity of the sample and the in-depth interviews provided a richness in the data critical to reflective thematic analytical process.^56,78^ However, it remains that the phenomenon of participating in specialized multidisciplinary rehabilitation interventions may not yet have been fully explored.^43^ It is our hope that with the detailed description provided the study design can be revisited with a larger sample in future research.

In light of our findings, several clinical implications and recommendations emerged. The program differences identified in this study, along with their unique strengths and weaknesses, will be helpful in supporting clinicians in their discussions with families about treatment options and are crucial in facilitating collaborative care decisions and establishing realistic treatment expectations. Recommendations for IIPT should carefully consider youths’ community peer support networks, their school attendance records, and future academic and career goals to minimize potential negative impacts. Our study also provides some insight into essential program components that contributed to achieving pain self-management. Interventions to support the development of better social functioning and peer relationships despite absences and limitations caused by chronic pain are required. Clear transition pathways should be developed and studied. Supportive mechanisms such as recommendations for ongoing intervention closer to home; collaboration with teachers, coaches, and other instructors; and cohort booster sessions should also be trialed. MMT programs could consider ways to further promote peer support and skill practice for both parents and youth with timely coaching support from clinicians. Attempts to minimize travel and time away from school and work for these families should be carefully considered. Finally, adherence to MMT recommendations, with focus on living well with pain instead of symptom reduction, could be further explore with youth and their families.

In conclusion, our findings helped to provide a detailed description of the treatment and posttreatment trajectories of youth with chronic pain enrolled in specialized multidisciplinary pain rehabilitation and the impacts of these programs on their lives. Not only were the benefits of these treatments highlighted but the detrimental effects were also unveiled, which have previously been unexplored. This information is imperative in supporting families in making care decisions and in improving clinical care pathways. Future research should focus on increasing access to these interventions, while addressing the perceived negative effects and impacts associated with them.

## Supplementary Material

Supplemental Material
