# Supplementary figures and images for: Youth and parent perceptions on participating in specialized multidisciplinary pain rehabilitation options: A qualitative timeline effect analysis
Source: Can J Pain. 2021 Feb 3;5(1):1–21. doi: 10.1080/24740527.2020.1858709 (PMC7951173; doi:10.1080/24740527.2020.1858709)

## SUPPLEMENTARY MATERIALS

### Appendix 1. Participant timeline example

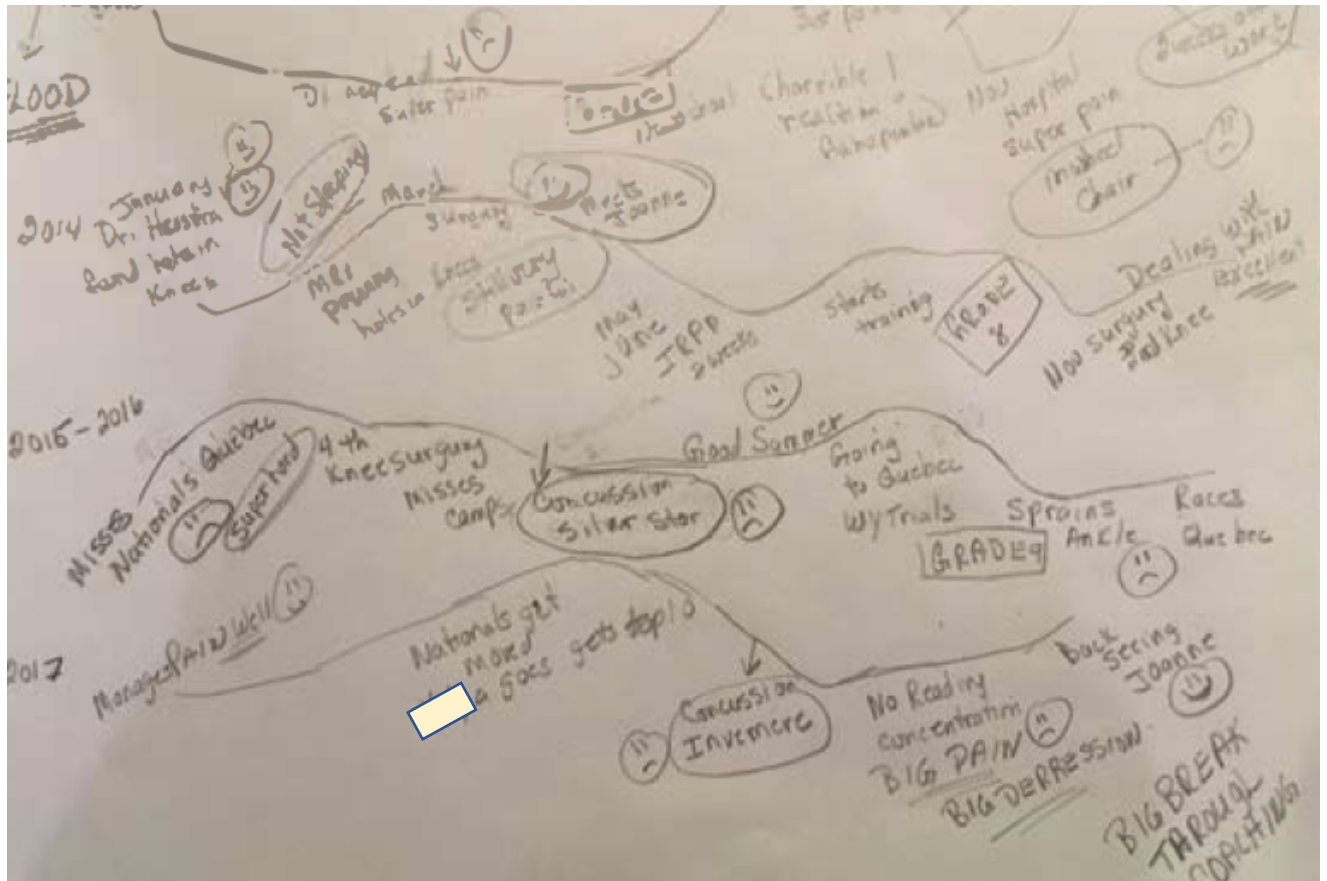

Supplement: Supplemental Material [file UCJP_A_1858709_SM8417.pdf]
